# Supplementary material for: Multi‐dataset identification of innovative feature genes and molecular mechanisms in keratoconus
Source: J Cell Mol Med. 2024 Sep 19;28(18):e70079. doi: 10.1111/jcmm.70079 (PMC11412914; doi:10.1111/jcmm.70079)
Supplement: Supplementary file 2 — Material S2. [file JCMM-28-e70079-s002.zip › 7.GSEA analysis/2.ATOH7/KEGG_GAP_JUNCTION.html]

Details for gene set KEGG\_GAP\_JUNCTION[GSEA]

|  || Dataset | ATOH7.group.cls#h\_versus\_l |
| Phenotype | group.cls#h\_versus\_l |
| Upregulated in class | h |
| GeneSet | KEGG\_GAP\_JUNCTION |
| Enrichment Score (ES) | 0.2971569 |
| Normalized Enrichment Score (NES) | 1.3522356 |
| Nominal p-value | 0.034623217 |
| FDR q-value | 0.3550524 |
| FWER p-Value | 0.932 |
Table: GSEA Results Summary

  

Fig 1: Enrichment plot: KEGG\_GAP\_JUNCTION      
 Profile of the Running ES Score & Positions of GeneSet Members on the Rank Ordered List

  

| SYMBOL | TITLE | RANK IN GENE LIST | RANK METRIC SCORE | RUNNING ES | CORE ENRICHMENT || 1 | PDGFD | na | 133 | 0.739 | 0.0563 | Yes |
| 2 | PDGFC | na | 274 | 0.635 | 0.1039 | Yes |
| 3 | TUBB2B | na | 400 | 0.580 | 0.1475 | Yes |
| 4 | PRKACB | na | 829 | 0.474 | 0.1737 | Yes |
| 5 | PLCB1 | na | 1718 | 0.367 | 0.1779 | Yes |
| 6 | PRKACG | na | 1784 | 0.361 | 0.2054 | Yes |
| 7 | PRKCB | na | 2281 | 0.324 | 0.2175 | Yes |
| 8 | GRM5 | na | 2585 | 0.304 | 0.2335 | Yes |
| 9 | GRM1 | na | 3125 | 0.274 | 0.2402 | Yes |
| 10 | PDGFB | na | 3642 | 0.250 | 0.2457 | Yes |
| 11 | SRC | na | 4033 | 0.235 | 0.2535 | Yes |
| 12 | MAP2K1 | na | 4240 | 0.228 | 0.2661 | Yes |
| 13 | HTR2A | na | 4344 | 0.224 | 0.2813 | Yes |
| 14 | DRD2 | na | 4638 | 0.213 | 0.2902 | Yes |
| 15 | ADCY3 | na | 5082 | 0.198 | 0.2936 | Yes |
| 16 | PRKG2 | na | 5483 | 0.186 | 0.2972 | Yes |
| 17 | ITPR1 | na | 7473 | 0.131 | 0.2504 | No |
| 18 | NRAS | na | 7693 | 0.126 | 0.2543 | No |
| 19 | PRKCA | na | 7938 | 0.120 | 0.2570 | No |
| 20 | GNAI1 | na | 9135 | 0.096 | 0.2304 | No |
| 21 | TUBA1B | na | 10363 | 0.076 | 0.2011 | No |
| 22 | GRB2 | na | 10441 | 0.075 | 0.2049 | No |
| 23 | ADCY8 | na | 10448 | 0.075 | 0.2108 | No |
| 24 | TUBA1A | na | 10692 | 0.071 | 0.2096 | No |
| 25 | TUBB | na | 10729 | 0.070 | 0.2142 | No |
| 26 | PRKACA | na | 12347 | 0.048 | 0.1715 | No |
| 27 | EGFR | na | 12485 | 0.046 | 0.1713 | No |
| 28 | PRKX | na | 12697 | 0.044 | 0.1688 | No |
| 29 | MAP3K2 | na | 12808 | 0.042 | 0.1690 | No |
| 30 | EGF | na | 13899 | 0.031 | 0.1401 | No |
| 31 | GJA1 | na | 13928 | 0.031 | 0.1419 | No |
| 32 | TUBB2A | na | 14289 | 0.028 | 0.1337 | No |
| 33 | MAPK1 | na | 14536 | 0.025 | 0.1287 | No |
| 34 | HRAS | na | 14559 | 0.025 | 0.1301 | No |
| 35 | GNAS | na | 14632 | 0.024 | 0.1300 | No |
| 36 | CSNK1D | na | 14704 | 0.024 | 0.1299 | No |
| 37 | RAF1 | na | 15219 | 0.019 | 0.1166 | No |
| 38 | MAP2K5 | na | 15356 | 0.018 | 0.1141 | No |
| 39 | MAPK3 | na | 16821 | 0.006 | 0.0724 | No |
| 40 | TUBA1C | na | 17643 | -0.001 | 0.0488 | No |
| 41 | ADRB1 | na | 17706 | -0.002 | 0.0471 | No |
| 42 | MAPK7 | na | 17787 | -0.002 | 0.0450 | No |
| 43 | GNA11 | na | 18372 | -0.007 | 0.0287 | No |
| 44 | TUBA3D | na | 18653 | -0.010 | 0.0214 | No |
| 45 | ITPR3 | na | 18764 | -0.011 | 0.0191 | No |
| 46 | HTR2B | na | 19789 | -0.020 | -0.0088 | No |
| 47 | GNAI2 | na | 19897 | -0.021 | -0.0102 | No |
| 48 | PRKG1 | na | 20059 | -0.022 | -0.0130 | No |
| 49 | GNAQ | na | 20613 | -0.028 | -0.0267 | No |
| 50 | MAP2K2 | na | 20653 | -0.028 | -0.0256 | No |
| 51 | ADCY9 | na | 20701 | -0.029 | -0.0246 | No |
| 52 | PLCB4 | na | 20753 | -0.029 | -0.0237 | No |
| 53 | TUBB4B | na | 21414 | -0.036 | -0.0398 | No |
| 54 | ADCY2 | na | 21650 | -0.039 | -0.0434 | No |
| 55 | TUBB6 | na | 22147 | -0.045 | -0.0541 | No |
| 56 | PDGFA | na | 22257 | -0.046 | -0.0536 | No |
| 57 | TUBB4A | na | 22530 | -0.049 | -0.0575 | No |
| 58 | SOS2 | na | 22550 | -0.049 | -0.0540 | No |
| 59 | TUBA3C | na | 23365 | -0.060 | -0.0726 | No |
| 60 | TUBB3 | na | 23857 | -0.068 | -0.0813 | No |
| 61 | PLCB3 | na | 24121 | -0.071 | -0.0831 | No |
| 62 | TJP1 | na | 24758 | -0.081 | -0.0948 | No |
| 63 | KRAS | na | 25083 | -0.087 | -0.0971 | No |
| 64 | ADCY5 | na | 25230 | -0.089 | -0.0941 | No |
| 65 | ADCY6 | na | 25322 | -0.091 | -0.0893 | No |
| 66 | TUBA4A | na | 25854 | -0.101 | -0.0964 | No |
| 67 | TUBB8 | na | 26179 | -0.108 | -0.0970 | No |
| 68 | ADCY7 | na | 26708 | -0.119 | -0.1026 | No |
| 69 | ITPR2 | na | 26843 | -0.122 | -0.0965 | No |
| 70 | SOS1 | na | 26852 | -0.122 | -0.0868 | No |
| 71 | LPAR1 | na | 27044 | -0.127 | -0.0820 | No |
| 72 | TUBA8 | na | 27102 | -0.128 | -0.0733 | No |
| 73 | DRD1 | na | 27257 | -0.132 | -0.0670 | No |
| 74 | ADCY4 | na | 27391 | -0.136 | -0.0598 | No |
| 75 | TUBB1 | na | 27653 | -0.142 | -0.0558 | No |
| 76 | GNAI3 | na | 28235 | -0.158 | -0.0597 | No |
| 77 | PLCB2 | na | 28336 | -0.160 | -0.0496 | No |
| 78 | PRKCG | na | 28978 | -0.178 | -0.0535 | No |
| 79 | CDK1 | na | 29770 | -0.203 | -0.0599 | No |
| 80 | ADCY1 | na | 30908 | -0.244 | -0.0729 | No |
| 81 | HTR2C | na | 30965 | -0.246 | -0.0545 | No |
| 82 | GJD2 | na | 32211 | -0.307 | -0.0655 | No |
| 83 | PDGFRB | na | 32781 | -0.349 | -0.0535 | No |
| 84 | TUBAL3 | na | 33288 | -0.393 | -0.0361 | No |
| 85 | GUCY1A2 | na | 33901 | -0.477 | -0.0150 | No |
| 86 | PDGFRA | na | 33905 | -0.477 | 0.0238 | No |
Table: GSEA details [plain text format]

  

Fig 2: KEGG\_GAP\_JUNCTION      
 Blue-Pink O' Gram in the Space of the Analyzed GeneSet

  

Fig 3: KEGG\_GAP\_JUNCTION: Random ES distribution      
 Gene set null distribution of ES for **KEGG\_GAP\_JUNCTION**
